# Pelvic Ultrasound in Diagnosing and Evaluating the Efficacy of Gonadotropin-Releasing Hormone Agonist Therapy in Girls With Idiopathic Central Precocious Puberty

**DOI:** 10.3389/fphar.2019.00104

**Published:** 2019-02-11

**Authors:** Hong-kui Yu, Xiao Liu, Jia-kun Chen, Shan Wang, Xian-yue Quan

**Affiliations:** ^1^Department of Radiology, Zhujiang Hospital, Southern Medical University, Guangzhou, China; ^2^Department of Ultrasonography, Shenzhen Children’s Hospital, Shenzhen, China

**Keywords:** ultrasonography, uterus, ovary, isosexual precocity, sexual characteristics

## Abstract

**Background and Objective:** Idiopathic central precocious puberty (ICPP) is characterized by early pubertal changes, the acceleration of growth velocity, and rapid bone maturation that often results in reduced adult height. Gonadotrophin-releasing hormone agonist (GnRHa) is currently considered to be an effective therapeutic agent. At present, GnRH stimulation test is adopted as a gold standard for the diagnosis of ICPP and the efficacy evaluation of GnRHa therapy. However, it is difficult to operate in practice due to the cumbersome procedures and multiple blood samples required. This study was conducted to establish the value of pelvic ultrasound in diagnosing ICPP and evaluating the efficacy of GnRHa therapy.

**Materials and Methods:** One hundred and twenty-two girls with ICPP (ICPP group) were enrolled in the study. Pelvic ultrasound and levels of luteinizing hormone (LH) and follicle-stimulating hormone (FSH) were examined before and after GnRHa therapy for 3 months. Eighty normal prepubertal girls were enrolled as the control group. The difference in pelvic ultrasound parameters between the ICPP group before GnRHa therapy and the control group was compared by independent-sample *t*-test, while paired *t*-test for ICPP group before and after GnRHa therapy. Receiver operating characteristic (ROC) curve was used to explore the optimal pelvic ultrasound parameters for diagnosing ICPP. Pearson correlation analysis was performed between the pelvic ultrasound parameters and serum sexual hormone level.

**Results:** The pelvic ultrasound parameters (length of the uterine body, anteroposterior diameter of the uterine body, transverse diameter of the uterine body, volume of the uterine body, uterine body-cervix ratio, length of the ovary, transverse diameter of the ovary, anteroposterior diameter of the ovary, volume of the ovary, number of increased follicles and maximum diameter of the follicle) in the ICPP group before GnRHa therapy were significantly larger than those of the control group (*P* < 0.05). All the above pelvic ultrasound parameters in the ICPP group were significantly decreased after GnRHa therapy compared with those before treatment (*P* < 0.05). The volume of the uterine body had the largest area under the ROC curve in differentiating between patients with ICCP and the control group. Pelvic ultrasound parameters were significantly correlated with serum sexual hormone levels (*P* < 0.05).

**Conclusion:** This study indicates pelvic ultrasound is a simple and reliable tool to diagnose ICPP and evaluates the efficacy of GnRHa therapy by dynamically observing the morphology of internal genitalia. The volume of uterine body was the best ultrasound parameter to distinguish patients with ICPP from normal girls.

## Introduction

Central precocious puberty is a condition characterized by early pubertal changes, the acceleration of growth velocity, and rapid bone maturation (Kaplowitz and Oberfield, 1999). The incidence of precocious puberty in children is on the rise year by year due to the influence of environment, food and social factors (Muir, 2006). Precocious puberty can be divided into central precocious puberty and peripheral precocious puberty according to pathogenesis. The most common type is idiopathic central precocious puberty (ICPP) (Chemaitilly et al., 2001). In girls, ICPP is caused by an increase in gonadotropin-releasing hormone (GnRH), due to early activation of the hypothalamic-pituitary-gonadal axis before the age of 8, which results in the development of the sexual organs and secondary sexual characteristics (Merke and Cutler, 1996). Without physical and mental maturity, the early onset of second sexual characteristics often cause fear, inferiority, anxiety and other adverse psychological problems, and can even lead to social problems and mental burdens to the parents. Additionally, due to accelerated skeletal growth and premature closure of the epiphysis, patients tend to be taller at the beginning, but shorter than normal adults when they grow up (Franceschi et al., 2010). Therefore, early diagnosis and treatment are essential (Rodriguez-Macias et al., 1999). Gonadotrophin-releasing hormone agonist (GnRHa) is currently considered to be an effective therapeutic agent (Latronico et al., 2016). Monitoring is required during GnRHa therapy to prevent ineffective administration caused by various factors. It is not convincing just to monitor blood levels of hormones such as luteinizing hormone (LH) and follicle-stimulating hormone (FSH) or physically examine the secondary sexual characteristics (Antoniazzi and Zamboni, 2004). The diagnosis of CPP and the efficacy of GnRHa therapy are both evaluated by GnRH stimulation tests as a gold standard so far (Lee, 1994; Choi et al., 2007). However, GnRH stimulation test is a cumbersome procedure with multiple invasive blood samplings, which are painful and expensive (Calcaterra et al., 2009).

Pelvic ultrasound can observe the size and structure of internal genitalia in girls, and can be used for the diagnosis and differential diagnosis of CPP, especially when the results of the GnRH stimulation test are ambiguous (de Vries et al., 2006; Badouraki et al., 2008; de Vries and Phillip, 2011a; Wen et al., 2018), and the operation is simple, non-invasive and easy to be accepted by children and their parents (Ziereisen et al., 2005). Up to now, a few literatures have reported the value of pelvic ultrasound in monitoring GnRHa therapy in girls with ICPP (Hall et al., 1986; Ambrosino et al., 1994; Jensen et al., 1998; de Vries and Phillip, 2011b), with limited cases (less than 30), and most of the literatures were published about a decade ago. There is also limited research data from China. Moreover, there is some controversy regarding which is the best parameter of pelvic ultrasound in evaluating the suppression of the hypothalamic-pituitary-gonadal axis. Ambrosino et al. suggested that ovarian volume changes can best reflect the efficacy of treatment (Ambrosino et al., 1994). However, de Vries and Phillip found that uterine parameters and absence of endometrial echo were better indicators of adequate suppression than ovarian parameters (de Vries and Phillip, 2011b). Therefore, this study was conducted to establish the value of pelvic ultrasound in diagnosing ICPP and evaluating the efficacy of GnRHa therapy.

## Materials and Methods

### Patients

All the girls enrolled in the ICPP group in this study had been clinically diagnosed as ICPP and received GnRHa treatment in our hospital from August 2013 to August 2018. The average age was (8.27 ± 0.72) years. ICPP was diagnosed according to the following criteria: (1) objective breast enlargement before 8 years of age, accompanied by the presence of one or more of the following findings: menses, pubic hair, accelerated growth velocity, (2) bone age that exceeds chronological age by at least 1 year, and (3) increased pubertal LH response (cutoff value ≥ 5 IU/L) on an immunoradiometric assay (IRMA) and LH-FSH ratio > 0.66 during GnRH stimulation test (Carel et al., 2009). The inclusion criteria included: patients diagnosed as ICPP, received GnRHa treatment, followed-up for at least 3 months, with intact clinical data. Patients with precocious puberty caused by tumor, organic disease, endocrine disease, simple premature breast development, a rare syndrome, misuse of contraceptives or other exogenous hormones were all excluded. Patients with poor ultrasound image quality or incomplete clinical data were also excluded. Pelvic ultrasound, GnRH stimulation test and sexual hormone levels were performed before GnRHa treatment and again 3 months after treatment.

Eighty normal prepubertal girls were enrolled in the control group. The controls were invited to participate in the study by the research staff. They were screened through studying their clinical history and through physical examination. All girls in the control group underwent pelvic ultrasound.

The body weights and heights of all subjects were measured, and the body mass indices (BMI) were calculated accordingly.

The ethics committee of our hospital approved the study and all parents of the patients gave informed written consent.

### Pelvic Ultrasound

Transabdominal pelvic ultrasound was performed using a GE LogiQ E9 ultrasound set (GE Medical Systems Inc., Ltd, United States), equipped with a 3–5 MHz convex-array broad-band transducer and a 9 MHz linear-array small parts transducer. Pelvic ultrasound was performed by experienced physicians with more than 3 years experience, who were blinded to the condition of the subjects. Oral intake of fluids was prescribed to all girls to obtain a moderately filled bladder, which serves as an acoustic window to view the pelvic organs and pushes the air-filled bowels aside. Scans of the ovaries and uterus were obtained carefully in both sagittal and transverse planes. The transducer was angled obliquely from multiple directions to improve visualization of the uterus and ovaries until optimal images were achieved.

After obtaining satisfactory ultrasound images of the uterus, the length, anteroposterior diameter and transverse diameter of uterus body, as well as the length of the cervix and the endometrial bilaminar thickness was measured. The uterus body-cervix ratio was calculated. The length, anteroposterior diameter and transverse diameter of ovaries were measured based on the optimal images of the ovaries. Increased follicle was defined as the diameter greater than 4 mm. The maximum diameter of the follicle and number of increased follicles were also recorded. Each data was measured three times and the average value was taken as the final measurement value. The uterine and ovarian volume was calculated by the formula: Volume = length × anteroposterior diameter × transverse diameter × 0.5233. There was no significant difference in the size of the bilateral ovaries (Wen et al., 2018). Therefore, the average of the bilateral ovaries was calculated for statistical analysis.

Ten randomly selected subjects underwent ultrasound examinations on the same occasion by two experienced operators who were unaware of the other’s results for reproducibility analysis.

### Measurement of Serum Sexual Hormones During the GnRHa Stimulation Test

The peak concentration of serum LH and FSH were measured before and after the GnRHa therapy. On an empty stomach in the morning, the patients were subcutaneously injected with Gonadorelin at a dose of 2.5∼3.0 μg/kg at the beginning of the GnRHa stimulation test. Blood samples (3 ml each time) were taken before (0 min) and after the test (30, 60, 90 min), and the levels of sexual hormones were determined by immunochemiluminescence (DX800; Beckman Coulter, Inc., CA, United States).

### The GnRHa Therapy

All children diagnosed as ICPP were treated with Triptorelin (produced by France’s Beaufort-Ipson Pharmaceutical Co., Ltd.), which was injected intramuscularly once every 4 weeks, 100 μg /kg each time. The maximum dosage was 3.75 mg. The dosage was adjusted according to the clinical symptoms of the children (Cirpan et al., 2013).

### Statistical Analysis

All statistical analyses were performed using SPSS version 19.0 (SPSS, Inc., Chicago, IL, United States). Measurement data were expressed as mean ± SD (standard deviation). The normal distribution test was conducted in the all pelvic ultrasound parameters. Independent-sample *t*-test was used to compare the difference of pelvic ultrasound parameters between the ICPP group before GnRHa therapy and the control group. Paired *t*-test was used to compare the difference in pelvic ultrasound parameters between ICPP group before and after GnRHa therapy. Receiver operating characteristic (ROC) curve was used to explore the optimal pelvic ultrasound parameters for distinguishing patients with ICPP from the normal population. Pearson correlation analysis was used to analyze the correlation between uterine and ovarian structural parameters and serum sex hormone levels before and after treatment. Two-tailed p values < 0.05 were considered statistically significant.

## Results

### General Information

A total of 122 girls were initially enrolled as ICPP group, and 3 cases of them were excluded due to poor ultrasound image quality, then 119 patients were eventually studied. There was no difference in age, height, weight and BMI between the ICPP group and the control group (*p* > 0.05) (Table 1). Compared with before GnRHa therapy, there was no significant difference in height and BMI after treatment (*p* > 0.05). Five patients had menstruation before treatment, and no menstruation existed in girls within the ICPP group after treatment. Before treatment, the Tanner stages of the breast in girls of the ICPP group were stage 2(*n* = 59), stage 3(*n* = 54) and stage 4(*n* = 6). After treatment, the breast development was inhibited to varying degrees, with Tanner stage 1(*n* = 14), stage 2(*n* = 77), and stage 3(*n* = 28).

**Table 1:** Comparisons of demographic data among ICPP patients before GnRHa therapy, ICPP patients after GnRHa therapy and normal controls.

Variables	Control subjects (*n* = 80)	ICPP before GnRHa therapy (*n* = 119)	ICPP after GnRHa therapy (*n* = 119)
Age (year)	8.41 ± 0.98	8.27 ± 0.72	8.52 ± 0.72^a^
Height (cm)	135.27 ± 4.43	135.02 ± 6.05	135.20 ± 6.04
Weight (kg)	33.41 ± 4.66	32.18 ± 6.11	33.10 ± 5.99
BMI (kg/m^2^)	19.18 ± 3.23	18.60 ± 5.79	18.52 ± 2.36


*Values are given as mean ± SD.*

*^a^Statistically significant compared with ICPP before GnRHa therapy.*

### LH Hormones and LH/FSH Ratio Before and After GnRHa Therapy

The LH level was significantly decreased after GnRHa therapy compared to before the therapy (22.48 ± 14.27 mIU/ml, vs. 1.06 ± 0.76 mIU/ml, *p* < 0.001). The LH/FSH was also significantly decreased after GnRHa therapy compared to before the therapy (1.45 ± 0.69, vs. 0.49 ± 0.27, *p* < 0.001).

### Pelvic Ultrasound

**FIGURE 1:**
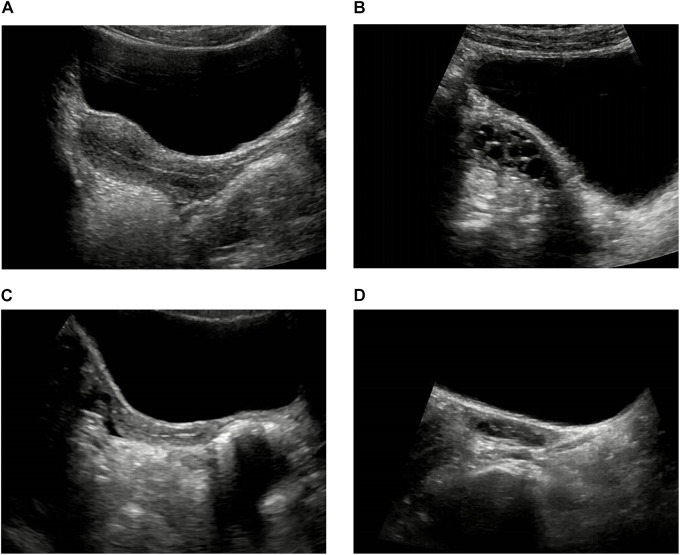
Typical sonograms of the uterus and ovaries of a ICPP patient before and after GnRHa therapy. **(A,B)** Before GnRHa therapy: the length, anteroposterior diameter, transverse diameter, volume of the uterine body and the ovary, uterine body-cervix ratio were all increased. There were three increased follicles in the ovary, and the maximum diameter of follicle was 8 mm. **(C,D)** After GnRHa therapy: the length, anteroposterior diameter, transverse diameter, volume of the uterine body and the ovary, uterine body-cervix ratio were all decreased. There was no increased follicle in the ovary.

The pelvic ultrasound parameters, including length of the uterine body, anteroposterior diameter of the uterine body, transverse diameter of the uterine body, volume of the uterine body, uterine body-cervix ratio, length of the ovary, transverse diameter of the ovary, anteroposterior diameter of the ovary, volume of the ovary, number of increased follicles and maximum diameter of the follicle, in the ICPP group before GnRHa therapy were significantly larger than those of control group (*P* < 0.05). All the above pelvic ultrasound parameters in the ICPP group were significantly decreased after GnRHa therapy compared with parameters before treatment (*P* < 0.05) (Table 2). Endometrial bilaminar thickening was observed in 6 ICPP patients. The thickness of endometrium was between 3 and 6 mm, and the thickened endometrium was not detected in any of the patients after GnRHa therapy. The typical sonograms of the uterus and ovaries of patients before and after GnRHa therapy are shown in Figure 1. The intraclass correlation coefficients between two observers of all of the above pelvic ultrasound parameters were 0.93–0.98 (*p* < 0.001 for all).

**Table 2:** Comparisons of uterine and ovarian parameters among ICPP patients before GnRHa therapy, ICPP patients after GnRHa therapy and normal controls.

Variables	Control subjects (*n* = 80)	ICPP before GnRHa therapy (*n* = 119)	ICPP after GnRHa therapy (*n* = 119)
Length of the Uterine body (cm)	2.02 ± 0.25	2.57 ± 0.47^a^	2.21 ± 0.28^b,c^
Anteroposterior diameter of the uterine body (cm)	1.10 ± 0.19	1.58 ± 0.42^a^	1.27 ± 0.25^b,c^
Length of the uterine body (cm)	2.02 ± 0.25	2.57 ± 0.47^a^	2.21 ± 0.28^b,c^
Anteroposterior diameter of the uterine body (cm)	1.10 ± 0.19	1.58 ± 0.42^a^	1.27 ± 0.25^b,c^
Transverse diameter of the uterine body (cm)	0.79 ± 0.18	1.21 ± 0.38^a^	0.91 ± 0.23^b,c^
Volume of the uterine body (cm^3^)	0.96 ± 0.49	2.94 ± 2.28^a^	1.43 ± 0.90^b,c^
Uterine body-cervix ratio	1.19 ± 0.08	1.37 ± 0.14^a^	1.25 ± 0.09^b,c^
Length of the ovary (cm)	2.26 ± 0.31	2.67 ± 0.37^a^	2.32 ± 0.29^b^
Transverse diameter of the ovary (cm)	1.06 ± 0.17	1.35 ± 0.26^a^	1.09 ± 0.16^b^
Anteroposterior diameter of the ovary (cm)	0.91 ± 0.17	1.15 ± 0.17^a^	0.93 ± 0.13^b^
Volume of the ovary (cm^3^)	1.19 ± 0.51	2.25 ± 0.88^a^	1.26 ± 0.41^b,c^
Number of increased follicles	1.19 ± 1.09	2.96 ± 0.84^a^	1.18 ± 1.16^b^
Maximum diameter of the follicle (mm)	3.45 ± 2.85	6.10 ± 1.18^a^	5.33 ± 0.89^b,c^


*Values are given as mean ± SD.*

*^a,c^Statistically significant compared with controls.*

*^b^Statistically significant compared with ICPP before GnRHa therapy.*

### ROC Analysis

**FIGURE 2:**
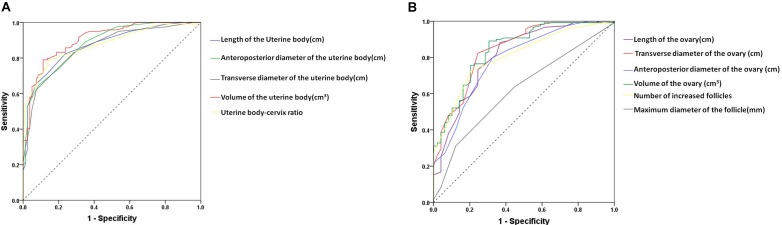
The receiver-operating characteristic curve for the pelvic ultrasound parameters to distinguish between patients with ICPP and controls. **(A)** uterine parameters, **(B)** ovary parameters.

The area under the ROC curve of pelvic ultrasound parameters are shown in Table 3 and Figure 2. The volume of the uterine body had the largest area under the curve in differentiating between patients with ICPP and normal girls. With the cutoff value of 1.01 cm^3^, the sensitivity and specificity in the diagnosis of ICPP were 91.6 and 68.7%, respectively.

**Table 3:** Receiver operative characteristic analyses of pelvic ultrasound parameters.

Parameter	Area under the curve	95% CI	Cutoff value	Sensitivity	Specificity
Length of the uterine body (cm)	0.871	0.822—0.920	2.15	82.4%	76.2%
Anteroposterior diameter of the uterine body (cm)	0.880	0.834—0.926	1.15	89.1%	65.0%
Transverse diameter of the uterine body (cm)	0.860	0.809—0.911	0.85	83.2%	71.2%
Volume of the uterine body (cm^3^)	0.904	0.863—0.945	1.01	91.6%	68.7%
Uterine body-cervix ratio	0.876	0.829—0.923	1.25	79.0%	85.0%
Length of the ovary (cm)	0.812	0.749—0.875	2.38	81.5%	68.7%
Transverse diameter of the ovary (cm)	0.871	0.821—0.921	1.18	82.4%	68.7%
Anteroposterior diameter of the ovary (cm)	0.825	0.766—0.884	1.03	79.8%	72.5%
Volume of the ovary (cm^3^)	0.871	0.822—0.921	1.43	89.1%	71.2%
Number of increased follicles	0.882	0.835—0.928	2.5	72.3%	87.5%
Maximum diameter of the follicle (mm)	0.622	0.533—0.712	5.5	63.9%	55.1%


### Correlation Between Sexual Hormone Levels and Ultrasound Parameters

Pearson correlation analysis showed that the pelvic ultrasound parameters were significantly correlated with serum sexual hormone levels (*P* < 0.05) (Table 4).

**Table 4:** Correlation of the pelvic ultrasound parameters with serum sexual hormone levels in ICPP group before and after GnRHa therapy.

Variables	LH (mIU/ml)		*LH/FSH*	
		
	*r*	*P*	*r*	*P*
Length of the uterine body (cm)	0.411	<0.001^∗^	0.380	<0.001^∗^
Anteroposterior diameter of the uterine body (cm)	0.354	<0.001^∗^	0.351	<0.001^∗^
Transverse diameter of the uterine body (cm)	0.378	<0.001^∗^	0.333	<0.001^∗^
Volume of the uterine body (cm^3^)	0.327	<0.001^∗^	0.311	<0.001^∗^
Uterine body-cervix ratio	0.327	<0.001^∗^	0.395	<0.001^∗^
Length of the ovary (cm)	0.324	<0.001^∗^	0.420	<0.001^∗^
Transverse diameter of the ovary (cm)	0.397	<0.001^∗^	0.373	<0.001^∗^
Anteroposterior diameter of the ovary (cm)	0.452	<0.001^∗^	0.448	<0.001^∗^
Volume of the ovary (cm^3^)	0.452	<0.001^∗^	0.486	<0.001^∗^
Number of increased follicles	0.547	<0.001^∗^	0.492	<0.001^∗^
Maximum diameter of the follicle (mm)	0.431	<0.001^∗^	0.337	<0.001^∗^


*^∗^Statistically significant.*

*LH, luteinizing hormone; FSH, follicle-stimulating hormone; r, Pearson correlation coefficient*.

## Discussion

In the present study, our findings showed that the level of serum sexual hormones decreased, which confirmed that GnRHa therapy was effective in girls with ICPP. A significant reduction in uterine and ovarian dimensions derived from pelvic ultrasound after GnRHa therapy was also discovered. The volume of uterine body had the highest discriminative ability to separate patients with ICPP from normal girls. The sensitivity and specificity in the diagnosis of ICPP were 91.6 and 68.7%, respectively, with the cutoff value of 1.01 cm^3^. The pelvic ultrasound parameters were significantly correlated with serum sexual hormone levels in the ICPP group before and after GnRHa therapy.

In this study, we found that the uterus, ovaries and the follicles were enlarged in the ICPP group before GnRHa therapy, which was consistent with the previous study (Bridges et al., 1995). Another earlier study determined that ovarian enlargement was an important piece of evidence for the diagnosis of central precocious puberty (King et al., 1993), but the sample size of the study was limited. The results of this study showed that the ovarian volume of the girls with ICPP was significantly larger than that of the control group, and the number of follicles and maximum follicle diameter both increased, demonstrating the characteristics of ovarian maturation. The ovarian maturation was promoted by the increased secretion of FSH, therefore, the increase in bilateral ovarian volume is an important indicator of ICPP and will be useful for its diagnosis. However, the results of our study showed that the volume of the uterine body was the best indicator for the diagnosis of ICPP. We consider that our findings were consistent with the general rule of uterine and ovary development. As the development of the uterus is influenced by the sexual hormones secreted by the ovary, the increase in the uterine volume indicates that the ovary has developed. Therefore uterine enlargement provides a better diagnostic index than ovarian enlargement. According to our study, with a cutoff value of 1.01 cm^3^ in the volume of uterine body to diagnose ICPP, the sensitivity and specificity are 91.6 and 68.7%, respectively. Wen et al. reported endometrial thickness was the best parameter for distinguishing CPP from normal girls in the 8–10 year interval, where a cutoff of 0.26 cm had a sensitivity of 76.92% and specificity of 100% (Wen et al., 2018). The cutoff values have a certain reference value for the diagnosis of CPP. However, as mentioned in previous studies (Eksioglu et al., 2013), there was a partial overlap between normally developed girls and CPP girls, and it could only be applied to girls of the age group involved in this study. Therefore, it is necessary to combine multiple ultrasound parameters with clinical manifestations and sexual hormone levels for the diagnosis of CPP.

### GnRHa Is Currently the Top Choice for the Treatment of ICPP

Gonadotropin-releasing hormone (GnRH), also known as luteinizing hormone-releasing hormone (LHRH), is a decapeptide hormone secreted by the hypothalamus (Latronico et al., 2016). GnRH is secreted by pulses from the hypothalamus into the pituitary portal system, stimulating the synthesis and secretion of pituitary LH and FSH, thereby regulating the secretion of sex hormones. GnRHa is a structural analog synthesized through modifying the molecular structure of GnRH. It can help the children achieve their expected height in adulthood, by eliminating the symptoms of precocious puberty and slowing down the maturity process of bone. GnRHa can block the receptors of GnRH as an extrinsic GnRH analog, then the pituitary gland no longer responds to normal GnRH and the hypothalamic-pituitary-ovarian axis is blocked, resulting in reduced secretion of the sex hormones. After intramuscular administration of the slow-released dosage form, the medicine was steadily released for 28 days after an initial release phase. The most widely used drugs are Triptorelin and Leuprorelin in long-acting depot preparations, which are considered to be more effective than daily doses (Tatò et al., 2001). Depot preparations have fewer compliance problems compared with daily subcutaneous and nasal spray preparations (Tuvemo et al., 2002). The drug used in this study was Triptorelin.

We can infer whether the hypothalamic-pituitary-gonad axis is activated or not through the changes of the ultrasound parameters, which can indirectly reflect the serum sexual hormone levels (Jensen et al., 1998). To the authors’ knowledge, this is the first comparative study, using large samples, studying the application of pelvic ultrasound and assessing the efficacy of GnRHa in the treatment of girls with ICPP. In our study, 122 girls with ICPP were enrolled, and 119 girls with complete data were eventually analyzed. Previous investigators suggested that the uterine and ovarian structural parameters decreased rapidly after GnRHa treatment in 3 months but decreased slightly with the prolonged treatment (de Vries and Phillip, 2011b). Therefore, our study focused on the evaluation of the parameters of uterus and ovaries and the serum sexual hormone levels before and after treatment in 3 months, then compared the changes accordingly.

Our results indicated that the pelvic ultrasound parameters in the ICPP group were significantly decreased after GnRHa therapy for 3 months compared with that before treatment, indicating that the stimulation effect on the uterus and ovaries induced by excessive sex hormones was well controlled. The morphology of uterus and ovaries was restored to different degrees through the therapy. The results indicated that the ovarian and uterine morphological changes measured by pelvic ultrasound in ICPP patients before and after GnRHa therapy can indirectly reflect the changes in hypothalamic-pituitary-gonadal axis function. However, the uterus parameters after GnRHa therapy were still significantly increased compared with those in the control group, while most ovarian parameters, except for the volume of the ovary and maximum diameter of the follicle, had no significant difference. In other words, after 3 months’ treatment, the ovaries shrink to normal size, while the uterus remains larger than normal. This might be attributable to the development of uterus being affected by ovarian hormones, therefore, the change in the uterus lags behind that of the ovaries. The pelvic ultrasound parameters were also consistent with the serum sexual hormone levels, demonstrating a good correlation. The LH peak level and the LH/FSH ratio in ICPP group were significantly increased under basic conditions. LH peak value and LH/FSH ratio were decreased after treatment compared to before treatment. After treatment, menstruation disappeared and the development of breast decreased in different degrees, however, there is no statistically significant change in height and BMI, which is consistent with a previous study (de Vries and Phillip, 2011b). This could be due to the fact that patients with ICPP tend to be taller at the beginning and shorter during the late phase, and patients in our study were at different stages of precocious puberty.

There were only six cases of endometrium thickening before treatment in the ICPP group in our study, and endometrium all disappeared after treatment. Some authors (de Vries and Phillip, 2011b) considered that the most significant response of treatment is the disappearance or reduction of endometrium. There were fewer cases with endometrial thickening in this study than in their study. The probable reason might be that patients in our study were in the primary stage of the disease with a relatively mild degree of development.

Ultrasound parameters were significantly correlated with serum sexual hormone levels before and after GnRHa therapy. The number of increased follicles had the best correlation with sexual hormone levels. This may indicate that the number of enlarged follicles is the most valuable parameter to reflect the changes after treatment. A few studies suggested that follicular development is not a good indicator of ovarian development (King et al., 1993). However, absence of visible cysts is a significant indicator of suppression (Ambrosino et al., 1994), and the number and size of stimulated follicles progressively approach those of the infantile ovary with suppression.

In brief, consistent with previous studies (Lekaemiri et al., 2014; Iannetta et al., 2015), our results indicated that pelvic ultrasound could dynamically monitor the therapeutic efficacy of GnRHa and provide reference for the clinical adjustment of follow-up treatment of ICPP. Pelvic ultrasonography provides a convenient and objective modality for monitoring pituitary-gonadal axis inhibition during ICPP therapy, which can lower the need for repetitive GnRH stimulation tests. It is helpful to confirm the efficiency of treatment. It could be a supplement of the GnRH stimulation test and more acceptable by ICPP girls and their parents. The advantages of pelvic ultrasound also include the ability to non-invasively and quickly evaluate the efficacy of the GnRHa therapy.

There were several limitations in our study, firstly that this was not a randomized controlled trial as the development stages of each patient were different. Secondly, although the sample size of this study was larger than most of previous studies, the age of patients in our study was concentrated between 6 and 10 years old. Normal reference values of uterus and ovary parameters for girls of all ages of puberty still need to be further explored. Thirdly, the follow-up time of girls with precocious puberty was short, and some were not followed up until drug withdrawal. The long-term application value of pelvic ultrasound in GnRHa therapy still needs to be further explored through prolonged follow-up time.

## Conclusion

In conclusion, pelvic ultrasound is a simple, non-invasive and reproducible method for the diagnosis of ICPP. Pelvic ultrasound is also a reliable method for the evaluation of the efficacy of GnRHa treatment. Pelvic ultrasound could serve as a promising tool for the clinical diagnosis and treatment in girls with ICPP.

## Author Contributions

H-kY and XL carried out analysis and calculations of data, and contributed to the writing of the manuscript. SW and J-kC contributed to data collection and management. X-yQ designed and coordinated the project, contributed to the discussion, supervised and reviewed the writing of the manuscript. All authors read and approved the final manuscript.

## Conflict of Interest Statement

The authors declare that the research was conducted in the absence of any commercial or financial relationships that could be construed as a potential conflict of interest.
